# Infiltrating Mast Cells Correlate with Angiogenesis in Bone Metastases from Gastric Cancer Patients

**DOI:** 10.3390/ijms16023237

**Published:** 2015-02-02

**Authors:** Michele Ammendola, Ilaria Marech, Giuseppe Sammarco, Valeria Zuccalà, Maria Luposella, Nicola Zizzo, Rosa Patruno, Alberto Crovace, Eustachio Ruggieri, Alfredo Francesco Zito, Cosmo Damiano Gadaleta, Rosario Sacco, Girolamo Ranieri

**Affiliations:** 1 Department of Medical and Surgical Sciences, Clinical Surgery Unit, University “Magna Graecia” Medical School, Viale Europa, Germaneto, Catanzaro 88100, Italy; E-Mails: michele.ammendola@libero.it (M.A.); sammarco@unicz.it (G.S.); valezy@libero.it (V.Z.); marilyn_luposella@live.it (M.L.); sacco@unicz.it (R.S.); 2 Surgery Unit, National Cancer Research Centre Istituto Tumori “Giovanni Paolo II”, viale Orazio Flacco 65, Bari 70124, Italy; E-Mail: eustachio.ruggieri@tiscali.it; 3 Diagnostic and Interventional Radiology Unit with Integrated Section of Translational Medical Oncology, National Cancer Research Centre, Istituto Tumori “Giovanni Paolo II”, viale Orazio Flacco 65, Bari 70124, Italy; E-Mails: ilariamare@tin.it (I.M.); c.gadaleta@oncologico.bari.it (C.D.G.); 4 Chair of Pathology, Veterinary Medical School, University “Aldo Moro” of Bari, Via Casamassima, Bari 70010, Italy; E-Mails: nicola.zizzo@uniba.it (N.Z.); rosavet@libero.it (R.P.); alberto.crovace@libero.it (A.C.); 5 Department of Emergency and Organ Transplantation (D.E.T.O.), Veterinary Medical School, Università “Aldo Moro”, Via Casamassima, Bari 70010, Italy; 6 Pathology Unit, ASL BA, Contrada Capo Scardicchio 92, Bari 70100, Italy; E-Mail: fazito@libero.it

**Keywords:** gastric cancer, bone metastases, mast cells, tryptase, anti-angiogenetic therapy

## Abstract

While gastric cancer is a well established angiogenesis driven tumor, no data has been published regarding angiogenesis stimulated by mast cells (MCs) positive for tryptase in bone metastases from gastric cancer patients (BMGCP). It is well established that MCs play a role in immune responses and more recently it was demonstrated that MCs have been involved in tumor angiogenesis. We analyzed infiltrating MCs and neovascularization in BMGCP diagnosed by histology. A series of 15 stage T_3-4_N_2-3_M_1_ (by AJCC for Gastric Cancer Staging 7th Edition) BMGCP from bone biopsies were selected. Tumour tissue samples were evaluated by mean of immunohistochemistry and image analysis methods in terms of MCs density positive to tryptase (MCDPT), MCs area positive to tryptase (MCAPT), microvascular density (MVD) and endothelial area (EA). A significant correlation between MCDPT, MCAPT, MVD and EA groups to each other was found by Pearson and *t*-test analysis (*r* ranged from 0.68 to 0.82; *p*-value ranged from 0.00 to 0.02). Our very preliminary data suggest that infiltrating MCs positive for tryptase may play a role in BMGCP angiogenesis, and could be further evaluated as a novel target of anti-angiogenic therapy.

## 1. Introduction

Gastric cancer is an important cause of cancer-related mortality [1,2]. At the time of diagnosis, the majority of the patients usually have unresectable or metastatic disease [1]. The most common sites of metastases are the liver and the peritoneum, but in the advanced stages, there may be metastases to any region of the body including bone [1,2,3]. Sometimes bone metastasis is observed in younger patients with poorly differentiated tumors and this condition is known to pursue a rapidly deteriorating clinical course [2]. Although gastric cancer is a well established angiogenic driven tumor and its metastatic process is supported by angiogenesis [4,5] no data have been published regarding the correlation between angiogenesis and mast cells (MCs) in bone metastases from gastric cancer patients (BMGCP). With special reference to MCs, they are widely distributed throughout the body, where they are positioned close to blood vessels and epithelial cell layers [6]. MCs are particularly abundant in locations that are exposed to the outside world, such as lung, gastrointestinal tract and skin [6,7]. Since they take up permanent residence in these locations, they are always on hand to respond to infections that most commonly occur during wounding [8]. Immunoglobulin E (IgE) receptors are present on the MCs membranes [8]. When IgE-coated antigens bind to surface receptors, MC degranulation occurs [9]. Increased MCs tryptase has a central role in inflammatory and immediate allergic reactions initiated by IgE [8]. In addition, MCs de-granulate tumor necrosis factor alpha (TNF-α) in response to bacterial products by an antibody-independent mechanism [8,9]. MCs release their preformed mediators when they encounter the complement anaphylatoxins C3a and C5a, and organisms that attack humans often produce exogenous factors [e.g., bacteria-derived adenosine diphosphate (ADP) and mite-derived proteases] that also induce the release of the MC’s granules constituents via different surface receptors [8]. MC de-granulation is also stimulated by the activation of its membrane tyrosine kinase the *c-Kit* receptor (CD-117) by means of the Stem Cell Factor (SCF) [9,10,11,12]. From another point of view, MCs have been identified as important players in tumor angiogenesis [13]. In a milestone study the involvement of host MCs in tumor angiogenesis and metastases was evaluated by comparing the angiogenic response of genetically mast-cell-deficient *W*/*W*_v_ mice and mast-cell-sufficient +/+ littermate mice to subcutaneous growing B16-BL6 tumors [13]. The angiogenic response was found to be lower in *W*/*W*_v_ mice than in +/+ mice. With special regard to the link between MCs and metastases it was demonstrated that fewer *W*/*W*_v_ mice than +/+ mice developed spontaneous lung metastases, and furthermore, that *W*/*W*_v_ mice exhibited fewer lung metastases per mouse [13]. Bone-marrow repair of the MCs deficiency restored the angiogenic response of *W*/*W*_v_ mice and also restored the incidence of hematogenous metastases to approach that of +/+ mice [13]. More recently, MCs have been involved in tumor angiogenesis of several human and pet malignancies [5,14,15,16,17,18,19,20,21,22,23,24,25,26,27]. It has been well demonstrated that MCs can secrete classical pro-angiogenic factors, including Vascular Endothelial Growth Factor (VEGF), Fibroblast Growth factor-2 (FGF-2) and Thymidine Phosphorylase (TP) [6,9,21,23]. Interestingly MCs can secrete other molecules such as tryptase and TNF-α that play a role in angiogenesis other than an immune response [28].

In particular, tryptase is one of the most powerful non classical pro-angiogenic factors [6]. It has been demonstrated that tryptase induces *in vitro* microvascular endothelial cell proliferation in the matrigel assay and displayed capillary growth *in vivo* on chick embryo chorioallantoic membrane, conversely suppressed by tryptase inhibitors [29,30]. Tryptase is also an agonist of the proteinase-activated receptor-2 (PAR-2) in vascular endothelial cells, stimulating their proliferation [31]. Tryptase plays a proteolytic activity degrading extracellular matrix components that in turn release matrix-associated growth factors such as metalloproteaes (MMPs) and plasminogen activators (PA) [32,33]. Tryptase also acts indirectly by activating latent MMPs [34] and PA, both key enzymes of proteolytic systems that contribute to the degradation of extracellular matrix components [35]. It is important to note that extracellular matrix degradation is a critical step in the early stages of angiogenesis as well as during invasion and metastasis of tumor cells [32,33,34,35]. Data from human studies demonstrated that MCs positive for tryptase increase in number and vascularization in a linear fashion in solid tumors, such as human malignant melanoma [36] endometrial carcinoma [37], breast cancer [15], gastric cancer [38], colorectal cancer [39], and pancreatic ductal adenocarcinoma [18]. Interestingly serum tryptase released from MCs has been described as a circulating predictive tumor marker in colorectal cancer and in breast cancer before and after surgical resection, when tryptase levels significantly decrease [7,12,15,16,27,39].

With special reference to gastric cancer, published results from our group and others suggest that primary gastric tumor angiogenesis is supported from MCs positive to tryptase [38,40,41]. However, no data has been published regarding MCs positive for tryptase and angiogenesis in bone metastases from gastric cancer patients (BMGCP). Here, we aim to correlate MCs density positive to tryptase (MCDPT), MCs area positive to tryptase (MCAPT), microvascular density (MVD) and endothelial area (EA) to each other in a series of BMGCP diagnosed by bone marrow biopsies. Obtained data are discussed in order to hypothesize a novel anti-angiogenic approach in this subset of metastatic patients [4,7,14,15,38,39].

## 2. Results

**Figure 1:**
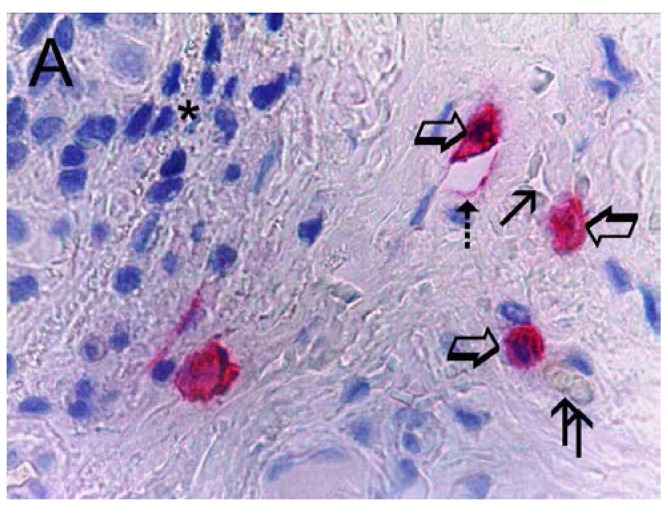
Bone metastasis from gastric cancer tissue section. Large arrows indicate red immunostained mast cells near microvessels. Single small arrow indicates the lumen of a microvessel with a single red blood cell in its lumen as an internal positive control. Double small arrow indicates a small microvessel with red blood cells in its lumen as an internal positive control. Discontinuous small arrow indicates a developing microvessel with a red immunostained endothelial cell positive to tryptase induced by the adjacent red immunostained mast cell positive to tryptase. The asterisk indicated a cluster of blue nuclei of gastric cancer cells. Maximum magnification of light microscopy 1000× in oil.

Immunohistochemical staining using the antibodies anti-CD31 and anti-tryptase showed that in highly vascularized cancer tissue, MCs positive to tryptase were well recognizable and were generally located in the perivascular position (Figure 1).

**Figure 2:**
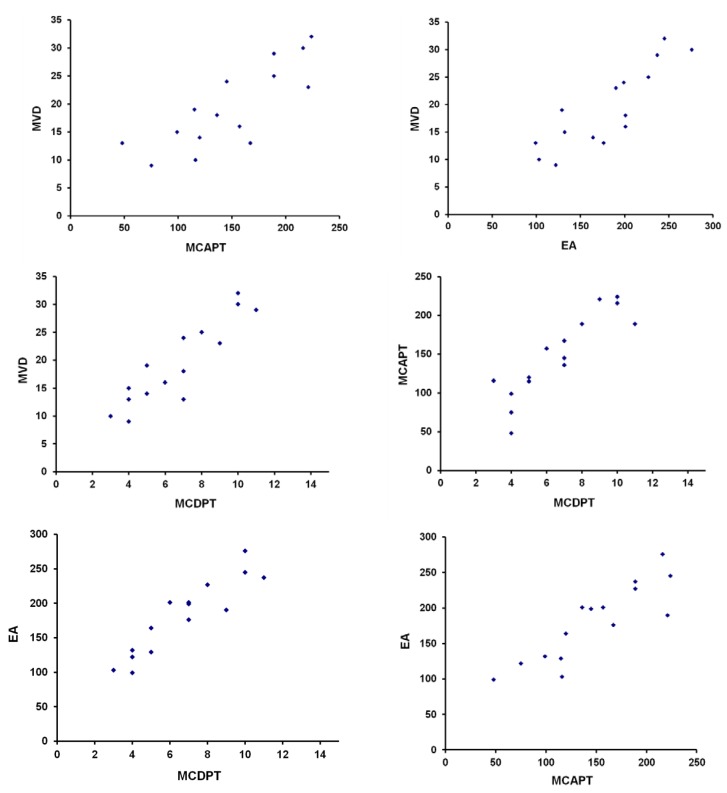
Each blue dot represents the linear correlation between MCDPT and MVD (*r* = 0.82, *p* = 0.00), MCDPT and MCAPT (*r* = 0.77, *p* = 0.01), MCDPT and EA (*r* = 0.73, *p* = 0.01), MCAPT and MVD (*r* = 0.68, *p* = 0.02), MCAPT and EA (*r* = 0.71, *p* = 0.02), between MVD and EA (*r* = 0.78, *p* = 0.01).

Mean values ± 1 standard deviations (SD) of all the tissue-evaluated parameters are reported in Table 1. With special reference to BMGCP, there was a significant correlation between MCDPT and MVD (*r* = 0.82, *p* = 0.00), between MCDPT and MCAPT (*r* = 0,77, *p* = 0.01), between MCDPT and EA (*r* = 0.73, *p* = 0.01), between MCAPT and MVD (*r* = 0.68, *p* = 0.02), between MCAPT and EA (*r* = 0.71, *p* = 0.02), and between MVD and EA (*r* = 0.78, *p* = 0.01) (Figure 2). G3 grading significantly correlated with bone metastasis (*p* = 0.001). No correlation concerning MCDPT, MCAPT, MVD, EA and the other main clinicopathological features was found.

**Table 1:** Mast cells (MCs) density positive to tryptase (MCDPT), MCs area positive to tryptase (MCAPT), microvascular density (MVD) and endothelial area (EA) means ± standard deviations as a function of bone metastases from gastric cancer patients (BMGCP) and primary tumor tissue respectively.

Tissue	MCDPT	MCAPT	MVD	EA
	400× (0.19 mm^2^)			
15 Bone metastases	6.66 ± 2.53	147.8 × 10^−2^ μ^2^ ± 53.66	19.33 ± 7.41	180.06 × 10^−2^ μ^2^ ± 54.29
21 Primary tumor	11.02 ± 4.73	223.63 × 10^−2^ μ^2^ ± 89.32	27.18 ± 9.82	231.71 × 10^−2^ μ^2^ ± 71.55

## 3. Discussion

From an immunological point of view, MCs positive to tryptase are involved in several inflammatory and immediate allergic IgE-mediated reactions [8,42]. MCs are players in the network of innate immunity in that they release TNF-α in response to bacterial products by means of an antibody-independent mechanism [8,9]. In the same manner, MCs can participate in tumor rejection, producing and releasing TNF-α and molecules such as interleukin 1 (IL-1), IL-4, and IL-6 that kill tumor cells [9]. For instance, the density of MCs in benign gastric ulcers was found to be much higher than in control subjects [42]. Furthermore, accumulation of MCs was also increased in well-differentiated gastric cancers when compared with controls, suggesting a mast cell intervention in the shift between inflammation and cancer [42]. Remarkably, poorly differentiated gastric adenocarcinomas showed lower density of MCs than well-differentiated adenocarcinomas [42].

**Figure 3:**
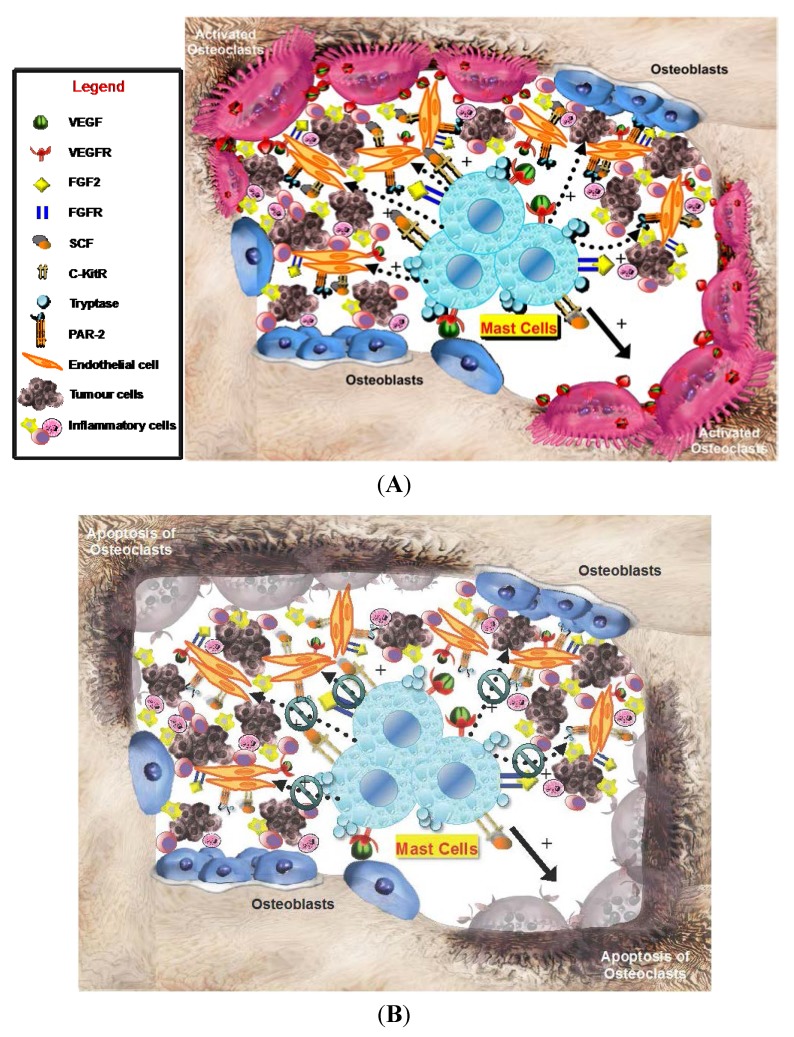
(**A**) The hypothetical crucial role of peritumoral MCs infiltrating surrounding gastric cancer cells in the bone microenvironment. MC degranulation, inducing the increase (see dotted arrows with +) of several pro-angiogenetic factors, such as VEGF, FGF2, SCF, and tryptase that bind or interfere with their specific receptors, expressed both by mast cells and endothelial cells, could promote osteoclast activation (see solid arrows), tumor angiogenesis, gastric cancer cell growth and; and (**B**) MCs targeting (see green symbols) in bone tumor microenvironment, *i.e.*, by means of tryptase inhibitors could represent a potential anti-angiogenetic strategy to inhibit osteoclast activation promoting their apoptosis and slow the progression of bone metastases. VEGF, Vascular Endothelial Growth Factor; VEGFR, Vascular Endothelial Growth Factor; FGF2, Fibroblast Growth Factor 2; FGFR, Fibroblast Growth Factor Receptor; SCF, Stem Cell Factor; *c-KitR*, *c-Kit* Receptor; PAR-2, Proteinase-Activated Receptor-2.

On the other hand, MCDPT is strictly correlated with the extent of pathological angiogenesis, occurring in several malignancies [5,7,11,12,14,15,16,17,18,19,20,21,22,24,27,38,39]. With special reference to gastric cancer, it has been already demonstrated that stage IV gastric carcinoma has a higher degree of vascularization than other stages, and that MCDPT increases in parallel with malignancy grade and is highly correlated with the extent of angiogenesis in primary tumor tissue [5,9,17,38,40,41]. However, based on our knowledge this is the first study that analyzed angiogenesis and infiltrating MCs positive to tryptase in bone tissue metastases from BMGCP. Our results showed a significant correlation between angiogenesis, evaluated in terms of MVD and EA, and infiltrating MCs in terms of MCDPT and MCAPT. These correlations suggest that, also in bone tissue, the development of metastases is supported through an angiogenic process stimulated by MCs positive to tryptase (Figure 3A). In bone tissue, MCs may be recruited and activated through SCF, the ligand of *c-Kit* receptor and by means of other growth factors such as, VEGF, FGF and TP, secreted by gastric cancer cells (Figure 3A) [4,28,29,30,43]. MCs contain several angiogenic factors including tryptase, VEGF, FGF, IL-8 and the above TNF-alpha that are all characterized by pro-angiogenic properties [4,11,32,42,44]. From them tryptase is the predominant and more abundant protease in MCs granules and it is a well demonstrated mitogen for both gastric cancer and endothelial cells [4,5]. Tryptase is one of the most powerful angiogenic mediators released by human MCs and it may be angiogenic via several mechanisms [14,30,32]. Blair *et al.* have demonstrated that direct addition of tryptase to microvascular endothelial cells cultured on matrigel caused a pronounced increase of capillary growth, which was suppressed by specific tryptase inhibitors, and directly induced endothelial cell proliferation in a dose-dependent fashion [30]. Similar results were obtained from Ribatti *et al.* on the chorioallantoic membrane *in vivo* assay [29]. Moreover, tryptase is an agonist of PAR-2, expressed on vascular endothelial and gastric cancer cells [31,32]. Activation of PAR-2 induces cell proliferation and release of IL-6 and granulocyte-macrophage colony stimulating factor, which, in turn, act as angiogenic molecules [31,32]. Taken together, these very preliminary data suggest that MCs positive to tryptase may play a role in bone metastasis angiogenesis from primary gastric cancer patients (Figure 3A), but they are not correlated with the main clinicopathological features. In this context, several tryptase inhibitors such as gabexate mesilate and nafamostat mesilate [14] might be evaluated in clinical trials for gastric cancer patients affected by bone metastases [1,4,45] (Figure 3B).

## 4. Patients and Methods

### 4.1. Ethics Statement

The study was approved by the Ethics Committee of “Mater Domini” Hospital, “Magna Graecia” University, Catanzaro (project identification code: 2011.61; date: 13 December 2011). According to the above Ethics Committee, a written consent was not needed. In fact, this is a retrospective observational study considering only deceased patients and thus their recruitment in the survey did not influence their treatment.

### 4.2. Patients

The retrospective analysis of records of more than 190 patients who died due to gastric cancer allowed the identification of patients with bone metastases. Briefly, patients with clinical suspected distant bone metastases staged as T_3-4_N_2-3_M_1_ on computed tomography and skeletal scan were identified. From these a series of 15 BMGCP (8% of global studied records) with confirmed histological bone metastases performed by biopsy were selected. Patients were staged according to the American Joint Committee on Cancer 7th edition (AJCC-TNM) classification and the World Health Organization classification (2000 version) was used for pathologic grading. The main clinicopathological features of patients are described in Table 2.

**Table 2:** Clinico-pathological features of 15 BMGCP patients.

Overall Series		Patients Number (*n* = 15)
Age	<65	9
	>65	6
Sex	Male	8
	Female	7
Primary tumour site	Cardia	2
	Lesser curvature	3
	Greater curvature	3
	Body and fundus	4
	Pyloric area	3
Stage (TNM) by AJCC classification	T_3-4_N_2-3_M_1_	15
Histology	Intestinal type	7
	Diffuse type	6
	Other	2
Grading	G3	12
	G2	3
Bone metastatic site	Spine	4
	Long bones	7
	Hip	4

The retrospective analysis of records also allowed the selection of a control series of 21 (11% of total studied records) gastric cancer patients with no distant metastases and staged as T_3-4_N_3_M_0_ that underwent primary tumour resection.

### 4.3. Immunohistochemistry

For the evaluation of MCDPT, MCAPT, MVD and EA a three-layer biotin-avidin-peroxidase system was utilized [15]. Briefly, 4 μm thick serial sections of formalin-fixed and paraffin-embedded of both bone biopsies and primary surgical removed tumor samples were deparaffinised. Then, for antigen retrieval, sections were microwaved at 500 W for 10 min, after which endogenous peroxidase activity was blocked with 3% hydrogen peroxide solution. Next, adjacent slides were incubated with the monoclonal antibody anti-CD31 (QB-END 10; Bio-Optica Milan, Milan, Italy) for the identification of endothelial cells diluted 1:50 for 1 h at room temperature and with the monoclonal antibody anti-tryptase (clone AA1; Dako, Glostrup, Denmark) for the identification of MCs positive to tryptase diluted 1:100 for 1 h at room temperature. The bound antibody was visualized using biotinylated secondary antibody, avidin-biotin peroxidase complex and fast red. Nuclear counterstaining was performed with Gill’s haematoxylin No. 2 (Polysciences, Warrington, PA, USA). Primary antibody was omitted in negative controls.

### 4.4. Morphometrical Assay

**Figure 4:**
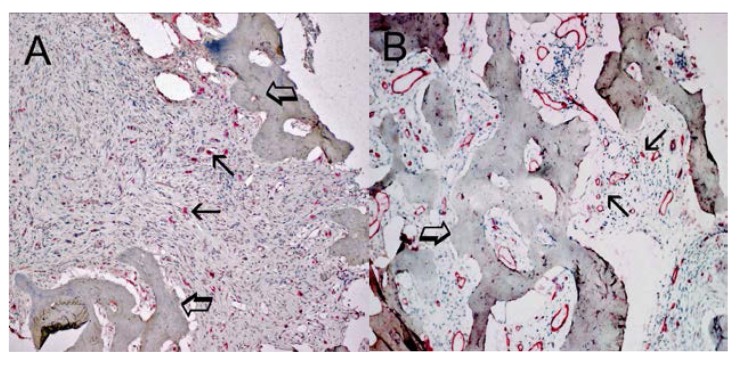
(**A**) Bone metastasis from gastric cancer tissue section. Many scattered mast cells positive to tryptase are seen as immunostained red. Small arrows indicate single mast cells. Large arrows indicate bone tissue. Low magnification: 100×; (**B**) Bone metastasis from gastric cancer tissue section. Many red immunostained microvessels are present. Small arrows indicate clusters of microvessels; note the small lumen. Large arrows indicate bone tissue. Low magnification: 100×.

**Figure 5:**
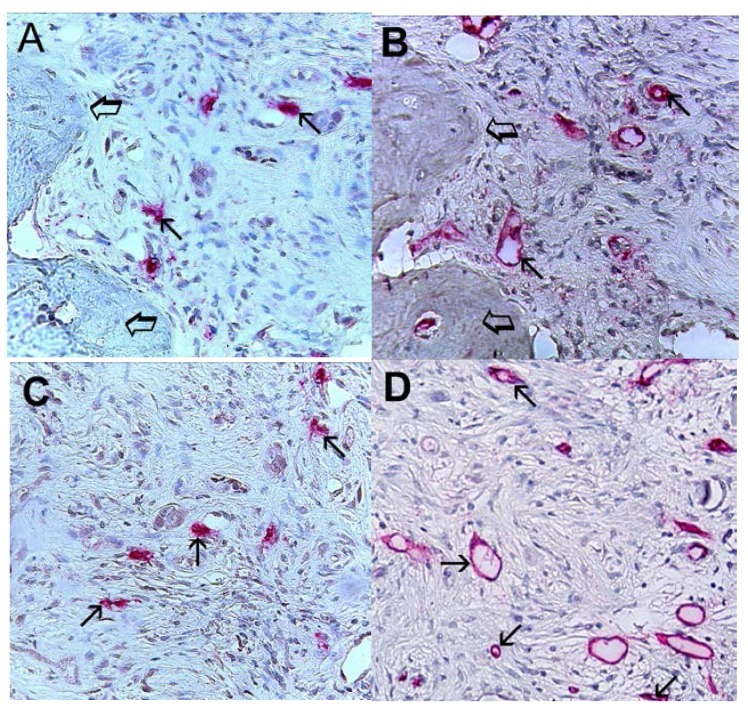
(**A**,**C**) Bone metastases from gastric cancer tissue sections. Small arrows indicate single red immunostained mast cells. Large arrows indicate bone tissue. Intermediate magnification: 400×; (**B**,**D**) Bone metastases from gastric cancer tissue sections. Small arrows indicate single red immunostained microvessels; note the small lumen. Large arrows indicate bone tissue. Intermediate magnification: 400×.

An image analysis system (Semiquantimet 400 Nikon, Tokyo, Japan) was employed. The five most vascularized areas (“hot spots”) were selected at low magnification and both MCDPT (Figure 4A) and individual vessel (Figure 4B) were counted at 400× magnification (0.19 mm^2^ area, Figure 5A–D) (GR, NZ and AFZ) [15]. Single red stained endothelial cells, endothelial cell clusters and microvessels, clearly separated from adjacent microvessels, tumor cells and other connective tissue elements were counted [15]. Areas of necrosis were not considered for counting. In serial sections each single MC positive to tryptase was counted. Single red stained endothelial cells and red stained MCs positive to tryptase were also evaluated in terms of immunostained area at 400× magnification (0.19 mm^2^ area) [15]. Finally morphological detail of both MCs positive to tryptase and endothelial cells was observed at 1000× magnification in oil (Figure 4).

### 4.5. Statistical Analysis

MCDPT MCAPT, MVD and EA mean values ± 1 SD were evaluated for each tissue sample and in all series of sections. The correlations between the above indexes and the clinicopathological features listed in Table 2 were analyzed by the Chi-square test. Linear correlations between MCDPT, MCAPT, MVD and EA groups to each other were quantified by means of the Pearson’s correlation coefficient (*r*) in biopsies from BMGCP. Correlation among MCDPT, MCAPT, MVD, EA groups and the main clinicopathological features were analysed by chi-square test. In all analyses, *p* < 0.05 was considered significant. All statistical analysis were performed with the SPSS statistical software package (SPSS, Inc., Chicago, IL, USA).
